# Characteristics of acute congestive heart failure with normal ejection fraction and less elevated B-type natriuretic peptide

**DOI:** 10.1186/1471-2261-9-2

**Published:** 2009-01-24

**Authors:** Ken Shimamoto, Natsuha Koike, Kiyoko Mizuochi, Miho Honma, Yufuko Kasai, Akiko Sakai, Etsuko Fujita, Masatoshi Kawana

**Affiliations:** 1 Department of Cardiology, Tokyo Women's Medical University Aoyama Hospital, Tokyo, Japan; 2 Department of Cardiology, Tokyo Women's Medical University Institute of Geriatrics, Tokyo, Japan

## Abstract

**Background:**

Heterogeneity in B-type natriuretic peptide (BNP) levels, especially among individuals with acute heart failure with normal left ventricular ejection fraction (HFNEF), can cause confusion in interpreting results. We investigated the characteristics of cases of acute HFNEF with only modestly elevated BNP.

**Methods:**

One hundred forty-two patients with acute or acute exacerbation of chronic HFNEF were divided into two groups by BNP level: BNP < 100 pg/ml (NB group, n = 45) and BNP ≥ 100 pg/ml (B group, n = 97). We compared clinical findings, echocardiography results, and neurohormonal factors between these two groups.

**Results:**

In the NB group, a history of open-heart surgery (OHS) was more frequent (71% vs. 22%, p < 0.0001) and hypertension was less frequent (p = 0.0005). Left atrial diameter (LAd) was higher (p = 0.0026), while interventricular septal thickness, posterior wall thickness, relative wall thickness, left ventricular mass index were lower (p = 0.0005, p = 0.0225, p = 0.0114, p = 0.0051, respectively) in the NB group. In patients with HFNEF, a history of OHS remained an independent predictor of BNP level (< 100 pg/ml) after adjustment for hypertension, age, LAd, and interventricular septal thickness (odds ratio 3.6, p = 0.0252).

**Conclusion:**

We found associations between acute HFNEF with less elevated BNP and a history of OHS. In a patient suspected of HFNEF, a history of OHS is considered diagnostic evidence of presence of diastolic heart failure when plasma levels of BNP are less elevated.

## Background

Heart failure with normal left ventricular ejection fraction (HFNEF), also called heart failure with normal systolic function or diastolic heart failure, accounts for 40–50% of cases of heart failure. This condition is commonly observed in elderly women, particularly those with a history of hypertension, left ventricular hypertrophy, or diabetes mellitus. Congestive symptoms, although generally milder, do not differ from those in systolic heart failure (SHF), in which ejection fraction is reduced. Although HFNEF is generally associated with a more favorable prognosis than SHF, the mortality rate for HFNEF and SHF are nearly equivalent in the elderly. Diagnosis of HFNEF must be made quickly to enable appropriate treatment, but this is often difficult, particularly in elderly patients who have comorbidities such as chronic lung or heart disease. B-type natriuretic peptide (BNP) level is a useful marker not only in the diagnosis and determination of the severity of heart failure, but also in the assessment of treatment effects [1-4]. However, there is marked heterogeneity in BNP levels among subjects with heart failure. Although a BNP level of approximately 100 pg/mL is regarded as a criterion for the diagnosis of heart failure, the interpretation of BNP level with regard to HFNEF is unclear [5-8]. BNP level has recently been reported to be also elevated in conditions other than heart disease, such as sepsis and subarachnoid hemorrhage, and to be affected by factors such as tachycardia, thyroid hormone, glucocorticoid, endothelin, angiotensin II, and renal function [9]. Understanding of extracardiac factors affecting BNP level is crucial when BNP level is used for differentiating heart disease. On the other hand, BNP level does not increase in heart failure resulting from tamponade or constrictive pericarditis [10,11]. In addition, because BNP level generally tends to be lower in HFNEF compared to SHF [12,13], the effects of extramyocardial factors must be considered in the development of HFNEF. In this study, we investigated the characteristics of cases of HFNEF with only modestly elevated BNP during the acute phase.

## Methods

### Study sample

One hundred and forty-two cases of acute or acute exacerbation of chronic HFNEF seen in our department between 2003 and 2006 were studied. The present study was a cross-sectional observational study in which the diagnosis of heart failure was based on the Framingham Criteria [14]. In all cases, more than one cardiologist was asked to agree on the diagnosis and severity according to the Framingham Criteria and the New York Heart Association class. This investigation is in conformity with the principles outlined in the Declaration of Helsinki and was approved by the Ethical Committee of Tokyo Women's Medical University; all subjects gave their informed consent.

The determination of HFNEF was made from symptoms, signs, chest x-ray results, and ultrasonic echocardiography (UCG) criteria (ejection fraction ≥ 50%, abnormal ratio of early diastolic flow velocity to peak late atrial diastolic flow velocity, and deceleration time of peak early filling velocity) within 24 hr of congestive heart failure event. Patients were divided into two groups with respect to BNP levels: BNP < 100 pg/ml (NB group, n = 45) and BNP ≥ 100 pg/ml (B group, n = 97). Patients with acute or severe mitral or aortic valvular dysfunction, myocardial infarction and obvious pulmonary disease were excluded. Cardiac abnormalities in this study included the following conditions: hypertension, diabetes mellitus, atrial fibrillation (AF), hypertrophic cardiomyopathy, and mild to moderate valvular disease. We compared clinical findings, history of open-heart surgery (OHS), hypertension, diabetes mellitus, and dyslipidemia, chest radiograph and ECG findings, parameters from echocardiography, frequency of atrial fibrillation (AF) and moderate or severe tricuspid valve regurgitation (TR), neurohormonal factors and other prognostic factors such as serum creatinine, hemoglobin and serum sodium between the two groups.

In some cases, maximal thickness of the pericardium was assessed by computed tomography (B group, n = 58, NB group, n = 24). Measurement were performed without zooming the pictures, using an electronic caliper that was placed where the pericardium was clearly visible and with lesser variations in thickness, namely on the anterior surface of the heart in front of the right and left ventricles.

### Blood sampling

Venous blood samples were obtained after 30 min of supine rest on admission for radioimmunoassay measurement of B-type natriuretic peptide (BNP), A-type natriuretic peptide (ANP), plasma norepinephrine, and plasma aldosterone concentration (PAC) levels. BNP level was measured by standard radioimmunoassay (Shionoria BNP kit; Shionogi Co., Osaka, Japan) within 24 hours. Values of BNP < 43 pg/ml were considered normal.

### Echocardiography

All subjects underwent standard two-dimensional echocardiography with a commercially available system (Sonos 5500, Philips Medical Systems, Best, The Netherlands and Vivid 7, GE Medical System, Horton, Norway) using a multi-frequency MHz transducer. Cardiac function was evaluated by M-mode echocardiography guided by two-dimensional imaging; left atrial diameters (LAd), interventricular septal thickness (IVST), posterior wall thickness (PWT), left ventricular end-diastolic diameters (LVDd), end-systolic diameters (LVDs), and ejection fraction (EF) were measured. The ratio of peak early diastolic flow velocity to peak late atrial diastolic flow velocity (E/A), deceleration time of peak early filling velocity (Dct), and right ventricular systolic pressure (RVSP) were measured by Doppler imaging. Left ventricular mass index (LVMI) was measured using the method of Simone et al. [14], while relative wall thickness (RWT) was measured using the method of Daniels et al. [15,16].

In addition, as a sub-analysis, patients with a history of OHS were subdivided into B and NB group for comparison of UCG parameters, BNP levels, frequency of AF and TR and history of hypertension.

### Statistical analysis

Continuous variables are expressed as mean ± SD and median value. For the relationship between UCG parameters, neurohormone levels, blood pressure, heart rate, age, and other prognostic factors, Mann-Whitney's U test was performed. Differences between groups described by categorical variables were analyzed by the chi-square test for independence or Fisher's exact probability.

The independent diagnostic value of HFNEF without elevated BNP was assessed in multivariate logistic regression model for all variables that proved to be significant by univariate analysis in patients with elevated BNP and those with less elevated BNP. The following dichotomous or continuous variables were evaluated in the multivariate model: medical history of OHS and hypertension, LAd, IVST, age, and PAC. All p values less than 0.05 are regarded as having statistical significance.

## Results

### Clinical and Neurohormonal Characteristics

Table 1 summarizes medical therapy, clinical findings, chest radiograph and ECG findings, biochemical profiles and pericardial thickness subdivided according to the BNP levels. The subjects were 77 men and 65 women, ages 73.7 ± 9.9 years old (range, 33 – 93 years old). The B group (age, 74.8 ± 10.4 years old) was significantly older (p = 0.0054) than the NB group (age, 71.4 ± 8.3 years old). Although hypertension was more common (p = 0.0005) in the B group, no differences were observed for diabetes and dyslipidemia. According to the New York Heart Association classification, the severity of heart failure was class II in 113 patients and class III in 29 patients, with no intergroup differences. As for treatment drugs, the B group received significantly more angiotensin-converting enzyme inhibitors and angiotensin II receptor blockers, while the NB group received significantly more digitalis, diuretic drugs, and warfarin (Table 1).

**Table 1:** Clinical and neurohormonal characteristics of the patients.

	B group	NB group	p
n	97	45	
Age (median) (yrs)	74.8 ± 10.4 (77)	71.4 ± 8.3 (72)	0.0054*
Gender male n (%)	56 (58)	21 (47)	0.2186
Body mass index (kg/m^2^)	22.1 ± 3.7 (22.2)	20.8 ± 3.6 (20.7)	0.0796
Open-heart surgery n (%)	21 (22)	32 (71)	< 0.0001
Mitral prosthesis n (%)	10 (10)	22 (49)	< 0.0001
Postoperative period (median) (yrs)	19.7 ± 11.0 (19)	21.1 ± 9.3 (21)	0.6290*
Hypertension n (%)	56 (58)	12 (27)	0.0005
Diabetes mellitus n (%)	25 (26)	12 (27)	0.9103
Dyslipidemia n (%)	22 (23)	4 (8)	0.0617
AngiotensinII receptor blockers/angiotensin- converting enzyme inhibitors n (%)	74 (76)	24 (53)	0.0067
Digitalis n (%)	21 (23)	23 (55)	0.0004
Calcium channel blockers n (%)	32 (36)	12 (29)	0.4245
β blockers n (%)	35 (39)	9 (21)	0.0426
Warfarin n (%)	37 (42)	29 (69)	0.0030
Diuretics n (%)	55 (61)	37 (88)	0.0020
Spironolactone n (%)	34 (38)	26 (62)	0.0094
NYHA II n	77	36	0.9321
III n	20	9	
Dyspnea n (%)	84 (88)	40 (91)	0.7752
General malaise n (%)	9 (9)	14 (31)	0.0018
Extra sound n (%)	8 (9)	1 (2)	0.2711
LV distension n (%)	33 (41)	22 (54)	0.1948
Hepatomegaly n (%)	32 (33)	24 (53)	0.0244
Peripheral edema n (%)	50 (52)	21 (47)	0.5486
Systolic blood pressure (median) (mmHg)	136.2 ± 23.6 (134.0)	134.4 ± 17.2 (132.0)	0.6778*
Diastolic blood pressure (median) (mmHg)	74.2 ± 14.8 (72.0)	72.3 ± 13.2 (70.0)	0.2300*
Heart rate (median) (bpm)	81.3 ± 66.5 (72.0)	74.4 ± 17.0 (72.0)	0.6566*
ANP (median) (pg/ml)	122.5 ± 185.4 (82.0)	29.0 ± 59.6 (15.0)	< 0.0001*
BNP (median) (pg/ml)	380.7 ± 259.1 (318.0)	59.7 ± 27.1 (60.8)	
Plasma aldosterone (median) (ng/dl)	6.9 ± 5.7 (5.0)	17.6 ± 24.1 (10.8)	< 0.0001*
Plasma norepinephrine (median) (ng/ml)	0.45 ± 0.29 (0.40)	0.38 ± 0.20 (0.36)	0.4405*
Serum creatinine (median) (mg/dl)	1.07 ± 0.46 (0.94)	0.96 ± 0.48 (0.90)	0.2460*
Hemoglobin (median) (g/dl)	11.9 ± 2.1 (12.1)	11.8 ± 2.1 (12.3)	0.9163*
Serum sodium (median) (mEq/L)	140.2 ± 3.9 (141.0)	140.1 ± 8.6 (140.0)	0.1607*
Chest radiograph:			
Cardiothoracic ratio (%)	61.2 ± 10.0	66.0 ± 14.3	0.0484*
Congestion n (%)	23 (26)	3 (12)	0.1826
Effusion n (%)	44 (50)	23 (58)	0.4303
ECG:			
High voltage n (%)	10 (14)	4 (14)	> 0.9999
ST change n (%)	34 (48)	13 (43)	0.6750
Intraventricular conduction disturbance			
n (%)	18 (21)	10 (28)	0.4366
Atrial fibrillation n (%)	47 (57)	29 (73)	0.0854
Pericardial thickness (median) (mm)	2.4 ± 1.1 (2.1)	2.9 ± 1.9 (2.0)	0.5886*

Chi-square test for independence or Fisher's exact probability, *Mann-Whitney's U test

**Figure 1:**
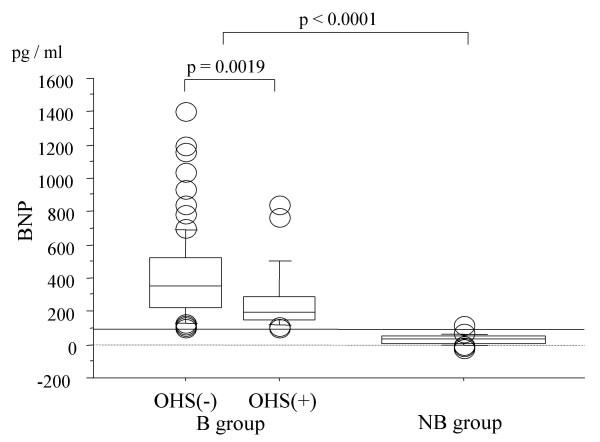
**Comparison of level of B-type natriuretic peptide (BNP) in B group between with and without a history of open-heart surgery (OHS). Patients who had a history of OHS had a significantly lower BNP level**. The boxes represent the 25th, 50th(median), and 75th percentiles, and the whiskers indicate the 10th and 90th percentiles.

The rate of exertional breathlessness was high and extrasounds were low in both groups, with no significant differences. However, the NB group had higher rates of general malaise (p = 0.0018) and hepatomegaly (p = 0.0244). The NB group also had a higher rate of history of OHS (71% vs. 22%; p < 0.0001), with a mean postoperative period of 21.1 years. The B group had a mean postoperative period of 19.7 years, demonstrating no significant differences from the NB group. In the NB group (n = 31), surgery involved mitral valve replacement (MVR; n = 13), mitral valve plasty (n = 1), double valve replacement (DVR) [mitral valve replacement + aortic valve replacement (MVR+AVR)] (n = 9), open mitral commissurotomy (OMC) (n = 6), and coronary artery bypass graft (CABG; n = 2). In the B group (n = 21), surgery involved MVR (n = 8), AVR (n = 3), DVR (MVR+AVR; n = 2), OMC (n = 1), atrial septal defect (ASD; n = 3), and CABG (n = 4). Especially, the NB group also had a higher rate of mitral prosthesis (49% vs. 10%; p < 0.0001),

No differences were observed for frequencies of high voltage, ST segment abnormality, intraventricular conduction delay, and AF on electrocardiography. Although cardiothoracic ratio on chest radiograph was greater in the NB group, no differences were observed for the frequencies of pulmonary congestion and pleural effusion. No intergroup differences were observed for mean heart rate and systolic and diastolic blood pressures on admission. The pericardial thickness in NB group tended to be thicker than that in NB group (2.4 ± 1.1 mm for B group versus 2.9 ± 1.9 mm for NB group, p = 0.5886). No clear calcified lesions were observed on computed tomography in patients with HFNEF with less elevated BNP. The NB group had a significantly lower ANP level (p < 0.0001) and a significantly higher PAC level (p < 0.0001). The B group with a history of OHS had a significantly lower BNP level than that without a history of OHS (p = 0.0019)(Figure 1).

### Echocardiographic findings

**Figure 2:**
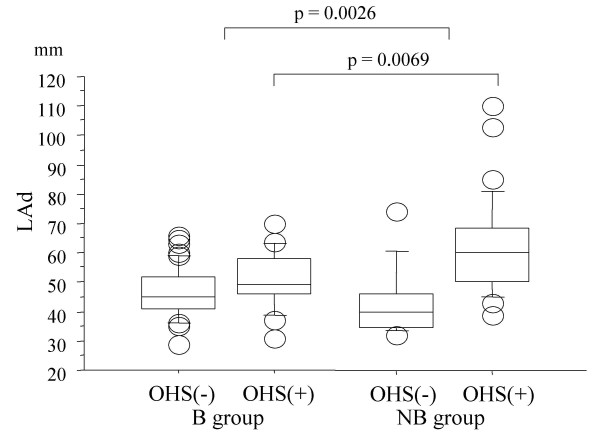
**Comparison of left atrial diameters (LAd) between B group and NB group**. Patients in the NB group who had a history of open-heart surgery had marked left atrial enlargement. The boxes represent the 25th, 50th (median), and 75th percentiles, and the whiskers indicate the 10th and 90th percentiles.

The echocardiographic parameters are shown in Table 2. No significant intergroup differences were observed for EF, LVDd, LVDs, RVSP and frequency of TR. The median values of IVST, PWT, LVMI and RWT were significantly higher in B group compared to NB group (p = 0.0005, p = 0.0225, p = 0.0051, p = 0.0114, respectively), indicating greater degree of LV hypertrophy in the B group. The NB group had a larger LAd (p = 0.0026) (Figure 2). Patients in the NB group who had a history of OHS also had marked left atrial enlargement (Figure 2). There was no significant difference in the frequency of moderate or severe TR between the two groups.

**Table 2:** Echocardiographic findings

	B group	NB group	p
LAd (median) (mm)	47.5 ± 8.6 (46)	56.5 ± 17.2 (53)	0.0026
IVST (median) (mm)	11.8 ± 2.9 (11)	10.0 ± 2.7 (10)	0.0005
PWT (median) (mm)	11.3 ± 2.5 (11)	10.4 ± 2.6 (10)	0.0225
LVDd (median) (mm)	47.1 ± 5.9 (49)	47.3 ± 6.3 (46)	0.4797
LVDs (median) (mm)	30.5 ± 5.2 (31)	29.6 ± 5.4 (30)	0.1566
EF (median) (%)	65.7 ± 8.5 (66)	67.1 ± 6.8 (67.5)	0.3042
RWT (median)	0.49 ± 0.13 (0.48)	0.44 ± 0.13 (0.42)	0.0114
LVMI (median) (g/m^2.7^)	73.1 ± 26.1 (70.1)	60.7 ± 24.8 (55.1)	0.0051
E/A (median)	1.3 ± 0.6 (1.2)	1.2 ± 0.5 (1.1)	0.7499
Dct (median) (ms)	213.3 ± 80.4 (185)	235.0 ± 79.4 (230)	0.3367
RVSP (median) (mmHg)	48.2 ± 14.6 (45)	44.7 ± 12.5 (41.5)	0.3035
TR (%)	24 (25)	15 (33)	0.2907*

Mann-Whitney's U test, * Chi-square test for independence or Fisher's exact probability

LAd: left atrial diameters, IVST; interventricular septal thickness, PWT; posterior wall thickness, LVDd: left ventricular end-diastolic diameters, LVDs: left ventricular end-systolic diameters, EF: ejection fraction, LVMI: left ventricular mass index, RWT: relative wall thickness, E/A: peak early filling velocity to atrial filling velocity ratio, Dct: deceleration time of peak early filling velocity, RVSP: right ventricular systolic pressure, TR: tricuspid regurgitation

### Sub-analysis of the OHS group

In the group with a history of OHS, the NB group had a larger LAd than the B group (p = 0.0069) (Figure 2). No significant differences were observed for left ventricular dimension, wall thickness, EF, LVMI and frequency of AF. However, the B group with had a higher frequency of hypertension than the NB group (p = 0.0093) (data not shown).

### Multivariate logistic regression analysis

Regarding logistic regression analysis, history of OHS, hypertension, IVST, LAd, age, and PAC were shown to be significant in univariate analysis, while only a history of OHS was shown to be significant in multivariate analysis (Table 3). The model showed that a history of OHS was strong independent predictor of HFNEF with less elevated BNP.

**Table 3:** Multiple logistic regression analysis of factors used for differentiating between patients with elevated BNP and those with less elevated BNP

Analysis	Predictor	Odds Ratio (95% CI)	p
Univariate	History of open-heart surgery	8.91 (3.98–19.9)	< 0.0001
	IVST	0.77 (0.66–0.91)	0.0018
	LAd	1.06 (1.03–1.10)	0.0005
	History of hypertension	0.27 (0.12–0.58)	0.0008
	PAC	1.16 (1.05–1.18)	0.0002
	
Multivariate	History of open-heart surgery	3.60 (1.17–11.03)	0.0252
	IVST	0.99 (0.80–1.21)	0.8838
	LAd	1.02 (0.98–1.07)	0.3743
	History of hypertension	0.36 (0.12–1.08)	0.0683
	PAC	1.06 (0.99–1.13)	0.1059

The odds ratio reflects the odds for patients with the characteristic in question as compared with those without the characteristic. CI denotes confidence interval

LAd; left atrial diameters, IVST; interventricular septal thickness, PAC; plasma aldosterone

## Discussion

The present findings suggest a relationship between HFNEF with less elevated BNP and history of OHS. Furthermore, in the B group, patients who had a history of OHS had a significantly lower BNP level. HFNEF with less elevated BNP was associated with a lower rate of history of hypertension as well as low values for left ventricular hypertrophy parameters. Patients in the NB group who had a history of OHS had marked left atrial enlargement, which may indicate permanent atrial fibrillation and pericardial constriction. Pericardial involvement may be responsible for HFNEF with less elevated BNP.

### Predominant mechanism of BNP release

Brain natriuretic peptide is secreted from the ventricles in response to increased ventricular wall stress in addition to myocardial stretch. Because a clear correlation is generally observed between left ventricular end-diastolic pressure (LVEDP) and BNP levels, BNP is used in the differential diagnosis of heart failure [17]. Merisel et al. recommend a cutoff value of 100 pg/ml of BNP in the diagnosis of heart failure [5].

However, in constrictive pericarditis, BNP does not increase despite increases in LVEDP [18]. Therefore, BNP has been reported to be a useful marker for differentiating constrictive pericarditis from restrictive cardiomyopathy [10]. During pericardial constriction with no distensibility, which is caused by factors such as constrictive pericarditis, no increases in transmural pressure are observed despite increases in intracavity pressure or left ventricular filling pressure, and myocardial stretch is inhibited [11]. Natriuretic peptide is clinically known to increase following pericardiectomy as well as removal of pericardial fluid [18-21]. Pericardiectomy or pericardiocentesis causes significant reductions in intrapericardial pressures, significant increases in transmural pressures and wall tension, and myocardial stretch [19,21]. Thus, natriuretic peptide secretion is thought to be stimulated by myocardial stretch resulting from increased transmural pressure, rather than by increased intracavity pressure [20].

### Pericardial adhesion in patients with prior open-heart surgery

In developed countries, the most frequent causes of constrictive pericarditis are prior cardiac surgery, idiopathic pericarditis, and irradiation therapy [22]. Pericardial adhesion, which is commonly observed following OHS, constitutes a problem during repeat surgery and may be an extracardiac factor that contributes to heart failure [22,23]. Because the postoperative patients have a long postoperative period following pericardiotomy, pericardial stretch is thought to be limited. In these patients, heart failure may easily become evident due to factors such as increased heart rate, salt or water loading, renal dysfunction and infection. The diagnosis of constrictive pericarditis should be considered in patients presenting with unexplained right sided heart failure and normal ejection fraction after cardiac surgery [24].

### Atrial degeneration and BNP secretion

B-type natriuretic peptide is also expressed and secreted in atrial tissue, and is stored in atrial granules along with ANP [25,26]. BNP also increases in AF and has been reported to exceed 100 pg/ml [27]. Therefore, the use of BNP in the differential diagnosis of breathlessness caused by AF may result in overdiagnosis of heart failure [28]. The kinetics of atrial-derived BNP in heart failure has not yet been elucidated. Although ANP increases in AF, it has been reported to decrease over time, possibly due to degeneration of atrial muscle [29,30]. ANP and BNP are structurally similar and are both stored in secretory granules [31]. In patients with permanent AF and enlarged atrial size, BNP is thought to decrease in a similar manner to ANP due to degeneration of atrial tissue. In HFNEF with a long postoperative period following OHS, especially valve replacement, the net increase in BNP is thought to be small due to more permanent AF resulting in severe atrial damage.

### Study Limitations and Perspectives

In the present study, evidence for pericardial lesions was based only on history of OHS and tendency toward pericardial thickening. Although radiographic determination of pericardium thickness is often taken into account during the assessment of constrictive pericarditis, increased pericardial thickness dose not necessarily imply constriction. Conversely, constrictive pericarditis can occur in patients with normal pericardium thickness [23]. It should not be denied because of normal pericardial thickness demonstrated by radiographic imaging when clinical, echocardiographic, or invasive hemodynamic features indicate constriction [23].

Hemodynamic assessment using right heart catheterization was performed for only two patients. However, non-compliant patterns, which are regarded as characteristic findings in constrictive pericarditis, were also observed in restrictive cardiomyopathies such as right ventricular infarction and amyloidosis, and were thus not a definitive finding. Furthermore, no characteristic waveforms are thought to be associated with mild constrictive pericarditis and effusive pericarditis. For the definitive diagnosis of constrictive pericarditis, it is essential to clarify the presence of intracardiac and intrathoracic dissociation and the presence of ventricular discordance. Therefore, constrictive pericarditis is often difficult to diagnose requiring careful attention to detail during measurement of hemodynamics. Eventually, the diagnosis of constrictive pericarditis is often confirmed at surgery. Thus, a simple measurement of BNP may be useful for differentiating constriction from restriction in HFNEF. Further study is required to determine the involvement of pericardial lesions in HFNEF with less elevated BNP.

## Conclusion

Acute heart failure with less elevated BNP is associated with a history of open-heart surgery and marked atrial enlargement. No association is observed between the level of BNP and left ventricular function. Atrial degeneration and an extramyocardial factor, particularly pericardium, need to be taken into account in the interpretation of the pathophysiology of heart failure with less elevated BNP. In a patient suspected of HFNEF, a history of open-heart surgery (especially mitral prosthesis) is considered diagnostic evidence of presence of diastolic heart failure when plasma levels of BNP are less elevated.

## Competing interests

The authors declare that they have no competing interests.

## Authors' contributions

KS conceived and designed this study, carried out the data analysis and statistical analysis and drafted the manuscript. NK, KM, MH, YK, AS, and EF performed the collection and analysis of data. MK helped to draft the manuscript and added important intellectual content.

All authors read and approved the final manuscript.

## Pre-publication history

The pre-publication history for this paper can be accessed here:


